# Multibiomarker responses to polycyclic aromatic hydrocarbons and microplastics in thumbprint emperor *Lethrinus harak* from a South Pacific locally managed marine area

**DOI:** 10.1038/s41598-021-97448-4

**Published:** 2021-09-09

**Authors:** Rufino Varea, Andrew Paris, Marta Ferreira, Susanna Piovano

**Affiliations:** School of Agriculture, Geography, Environment, Ocean and Natural Sciences, The University of the South Pacific, Suva, Fiji

**Keywords:** Enzyme mechanisms, Environmental impact, Enzyme mechanisms

## Abstract

To determine the baseline threat of microplastics and polycyclic aromatic hydrocarbons (PAHs) in an important seafood fish from Vueti Navakavu locally managed marine area, a multibiomarker risk assessment was conducted on the thumbprint emperor fish *Lethrinus harak*. Condition factor, a measure of relative general health condition of fish, was significantly lower in samples from the wet season compared to the dry season but no significant differences were observed for hepatosomatic index, a measure of relative stored energy/nutrition, between seasonal groups. PAHs levels of four metabolites in emperor fish from Fiji waters are reported here for the first time; seasonal groups showed no significant differences, but all samples presented levels of biliary PAHs. Each specimen also contained at least one microplastic in its gastrointestinal system; fibres were the predominant form-type and ingestion levels showed that more than 80% of fragment sizes were below 1.0 mm. Biochemical responses were observed for ethoxyresorufin-*O*-deethylase and glutathione *S*-transferase biotransformation activity, oxidative stress (glutathione peroxidase and glutathione reductase activity; lipid peroxidation) and genotoxicity (micronuclei assay). Though there were no statistically significant differences found, there were biological significances that were important to note; relatively low levels of pollutant exposure and low levels of biochemical responses showed enzymes response in thumbprint emperor were as expected to their roles in the body. In this multibiomarker approach, the observation of pollutants presence and histopathological injuries are considered biologically relevant from a toxicological perspective and serve as a baseline for future pollution studies in seafood fishes in Fiji, with site differences and the inclusion of fish species comparison. We recommend adopting a suite of biomarkers in future regional biomonitoring studies to develop holistic baseline information for other marine settings in Fiji and other Pacific Island countries.

## Introduction

Marine pollution, in its many forms, poses a major threat to ocean life^1,2^. Pollutants in the marine environment are human-introduced chemicals or organically-sourced compounds that can influence the natural function or role of ecosystems and its inhabitants^3^. Two ubiquitous pollutants are microplastics (MPs) and polycyclic aromatic hydrocarbons (PAHs)^4,5^. MPs are described as plastic particles that have a size range between 0.01 and 5 mm^6,7^, while PAHs are organic compounds made of carbon and hydrogen, grouped into multiple aromatic rings and are primarily generated by incomplete combustion of organic materials^8^. MPs can spread across vast spaces of the ocean via currents and winds^9^, and are subject to progressive fragmentation due to mechanical abrasion, ultraviolet radiation, and biodegradation^10^. MPs transfer through the trophic food chain, bioaccumulating and biomagnifying in seafood, likely posing a risk to human health^11,12^. Specifically, the major route of human exposure to MPs is ingestion, which can lead to inflammatory lesions and immune disorders^13,14^. In Fiji, recent screenings have found MPs in seawater, sediments, and marine fishes^15–17^, while PAHs have not been studied yet^18^. However, studies elsewhere have found PAHs in different marine compartments like sediments^19,20^, water^21,22^ and biota^23,24^. PAHs represent a class of chemicals whose metabolites can exhibit toxicity even at low levels of exposure^25^; they bioaccumulate in marine bivalves, crustaceans and fishes^26^, and are subject to biomagnification in the food chain^23,27^. Some PAH metabolites are carcinogenic to humans^28^. PAHs are of interest for Pacific Island countries and are a priority area in the Pacific Regional Waste and Pollution Management Strategy 2016–2025, though no baseline data of these pollutants in Pacific Islands marine environment is available^18^. There is also a growing concern of MPs in Pacific Island seawaters, as highlighted by the Environmental Investigation Agency^29^ in 2020. MPs are recognised as a priority to address in the 2050 strategy for a Blue Pacific Continent.

The process of determining the presence or stages of effects of pollutants, like MPs and PAHs, in the environment or its inhabitants, is called environmental risk assessment^30^. Environmental risk assessments entail two approaches; environmental monitoring via chemistry surveillance^31^ and biomonitoring using biomarkers^32^. In Fiji, environmental monitoring has been used in some forms of environmental risk assessments, however, biomonitoring has yet to be applied^18^. The application of biomarkers in biomonitoring is useful for measuring a biochemical response of an animal when a pollutant causes a change to its biological state^33^. In general, these biochemical changes are responses occurring at the lower organismic levels; i.e., molecular, subcellular, cellular, histological^34^. Several biomarkers cover a range of measurable parameters for determining biological responses to marine pollution. For example, fish health can be evaluated with Fulton’s condition factor (K)^35^ and the hepatosomatic index (HSI), which are relative indications of general nutritional status and stored energy, respectively^36^. Both the K and the HSI of marine fishes are influenced by pollution exposure^37^—in particular, PAHs and MPs have been found to cause reduced K and HSI of marine fishes^38^. At the systemic level, there are biomarkers used to measure the activity of biotransformation enzymes. This includes cytochrome P450 enzyme such as the ethoxyresorufin *O*-deethylase (EROD) in phase I and the glutathione *S*-transferase (GST) in phase II^39^. Additionally, oxidative stress responses are measurable through antioxidant enzymes activity like the glutathione peroxidases (GPX)^40^, glutathione reductases (GR)^41^ and associated oxidative damages like lipid peroxidation (LPO)^42^. At the molecular level, the degradation of DNA integrity due to pollutants can be evaluated via the number of occurrences of deformed erythrocyte nucleotides as biomarkers of genotoxicity^43^.

Animals that are specifically selected for biomonitoring of pollutants in the environment are called sentinel species^44^. In particular, sentinel species are those animals that have measurable responses to the (class of) agents in question, including having a known habitat that overlaps the monitored area, are easily enumerated and captured, and have sufficient population size/density^45^. Some examples of marine sentinel species are the common periwinkle *Littorina littorea*^46^, the goose barnacle *Pollicipes pollicipes*^47^, the guri sea catfish *Genidens genidens*^48^, and the flathead grey mullet *Mugil cephalus*^49^. Continuous biological surveillance using sentinel species can be used to evaluate the effects of pollution in the marine environment^50^, but also to monitor the results of conservation and pollution mitigation efforts.

**Figure 1:**
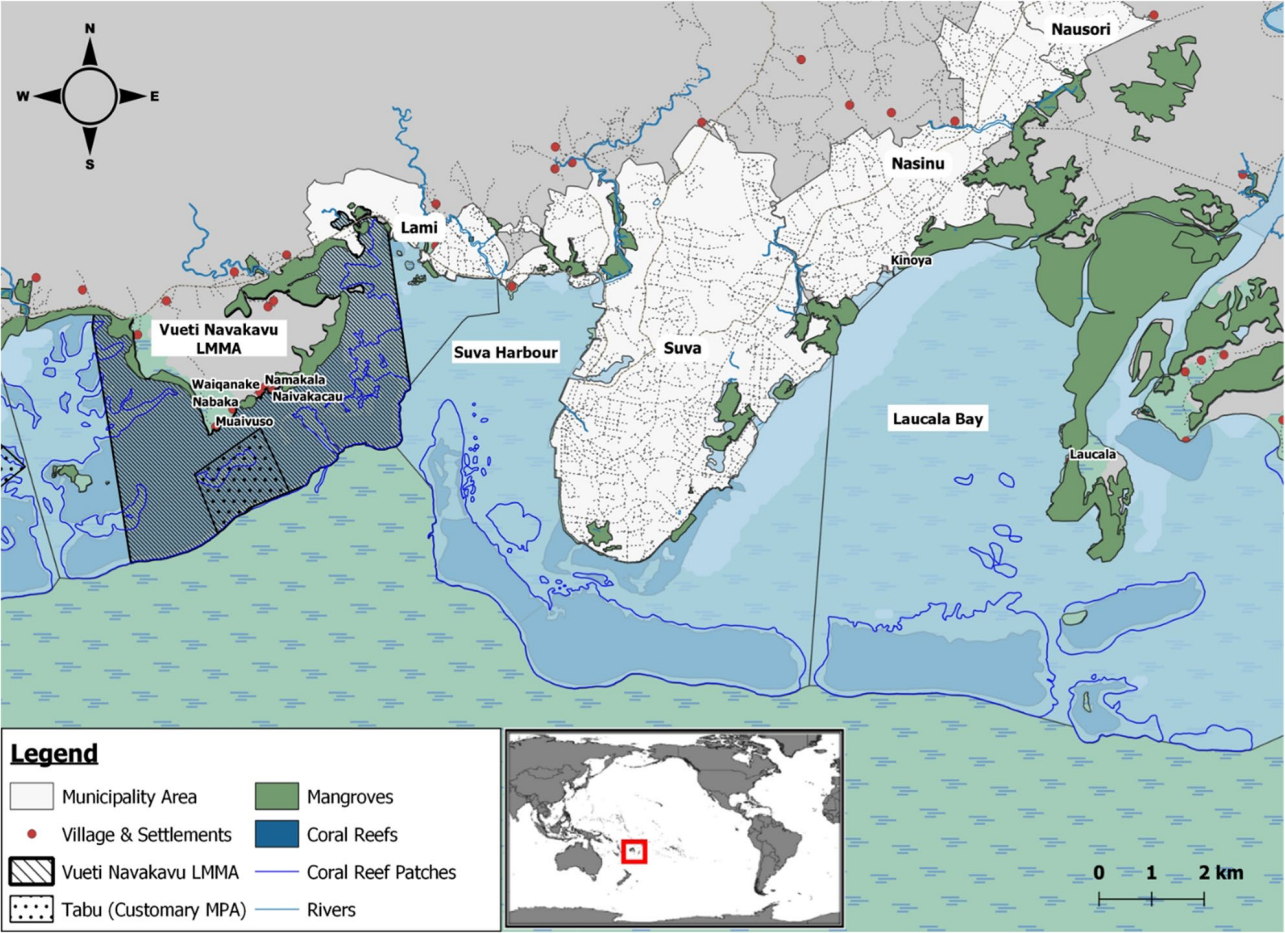
Vueti Navakavu locally managed marine area (LMMA) and its customary marine protected area (*tabu*) in Viti Levu, Fiji. Inset: location of Fiji within the Pacific Ocean. Maps produced with QGIS Development Team^57^; maritime boundaries from the Secretariat of the Pacific Regional Environment Programme^58^—PacGeo network.

In Fiji, the customary systems of managing inshore marine resources include a prohibition of fishing (*tabu*) that can be declared by local clans or villages (*mataqali*) over the area (*iqoliqoli*), where a village exerts its customary fishing rights^51^. Traditionally, this type of prohibition is used in special circumstances, such as the death of a chief, and for a few months only^52^. However, in recent times, *tabu* has been used also by customary resource owners to protect and preserve their local marine resources. About 465 *tabu* areas, covering ca. 1000 km^2^, are part of the Fiji locally managed marine area (LMMA) network^53^. Among them, is the 19.1 km^2^ “Vueti Navakavu” LMMA, which lies 5 km west from the capital of Fiji, Suva, and is managed by the clan of Navakavu (which is spread over the three villages Muaivuso, Nabaka, and Waiqanake, and the two settlements Naivakacau and Namakala) (Fig. 1). About 20% (3.8 km^2^) of the Vueti Navakavu LMMA is a no-take *tabu* zone. Vueti Navakavu LMMA was designated in 2002 after the villagers identified several threats affecting the area, among which chemical and solid waste pollution were mentioned^54^. Despite the conservation effort, MPs pollution has been found in the locally managed marine area^15,16^. In addition, ocean surface currents, influenced by the southeast tradewinds^55^, might have brought in pollutants from the close-by Suva Harbour, where the presence of MPs and heavy metals, for example, has already been reported^15,16,56^.

This study applies a biomonitoring ecotoxicological approach to environmental risk assessment in Vueti Navakavu LMMA in Fiji. Herein, PAHs exposure and presence, and abundance of MPs are quantified in the sentinel species thumbprint emperor (*Lethrinus harak*). Seasonality was considered when investigating the biological status of the thumbprint emperor to establish whether natural variation may influence pollution load into the marine environment, and thus affect the species interactions within (or changes to) the habitat.

## Methods

### Biological sampling

Sampling was performed from 100 to 250 m from the *tabu* area of Vueti Navakavu LMMA (Fig. 1) in April and July of 2017 and April and September of 2018, and it was performed to cover both the wet (November to April) and dry (May to October) tropical seasons. The thumbprint emperor was captured by local fishers with hook-and-line fishing gear. The live fish were placed in an 80 L portable tank filled with water from the fishing ground. Aeration was ensured by two submersible pumps (RS Electrical YS-702). In the village, the total weight and total length of each live fish were recorded using an analytical balance scale (precision: 0.1 g) and a measuring board (precision: 0.1 mm), respectively. Blood was extracted from the caudal vein of the live fish using a 21-gauge needle syringe and smeared onto a microscope glass slide to count for erythrocyte micronuclei formations^43^. The ethical sacrifice of the fish was then done by anaesthetising the fish in ice for 2 min, before severing a section in the vertebrae between the operculum and ray of the anterior dorsal fin using a scalpel blade^59^. The bile was extracted from the gall bladder using an insulin syringe for the fluorescence aromatic compounds analysis, then kept on ice until storage in a − 20 °C freezer. The liver was extracted and weighed. Five random sections of the liver were separated for the biochemical parameters and stored in liquid nitrogen until storage in a − 80 °C freezer.

### Biomarkers

Fulton’s condition factor was calculated as K = total weight/length^3^ × 100. The hepatosomatic index was calculated as HSI = liver weight/total weight × 100. The PAH metabolites were determined through fixed wavelength fluorescence (FF) screening method^60^ and achieved by diluting the bile (10:1000 µL) in 48% ethanol before being measured spectrofluorometrically (absorbance and fluorescence intensity; double monochromotors) in a multimode reader (Thermo Scientific™ Varioskan™ MIB#5250030) to determine the signals intensity ratios of four biliary PAH metabolite types; phenanthrene (FF_260/380_), naphthalene (FF_290/335_), 1-hydroxypyrene (FF_341/383_), and benzo[a]pyrene (FF_380/430_)^61,62^. The multimode instrument reader measured at a dynamic wavelength range (emission: 200–1000 nm; excitation 5 nm and 12 nm/12 nm) with an accuracy of 0.003 Abs or ± 2%, at 200–399 nm (0–2 Abs) and 0.003 Abs or ± 1%, at 400–1000 nm (0–3 Abs), which was within the required spectrofluorometric parameters for the fluorescent aromatic compounds (FACs) analysis^63^. The quality assurance and quality control for the four biliary PAH metabolites included analytical standards for each of the PAH metabolites measured, calibration curves, continuing calibration standards, and method blanks in accordance with the technical guidelines described by the International Council for the Exploration of the Sea^60,64^. To assess the activity of biochemical analysis of EROD, the liver was homogenized in ice-cold buffer (50 mM Tris–HCL, pH 7.4, 0.15 M KCl)^65^. The S9 fraction of the hepatic tissue was homogenized^66^. The EROD activity was evaluated fluorometrically^67^. GST activity was determined by a substrate artificial 1-chloro-2, 4 dinitrobenzene, which was conjugated by GST^68^. GPX activity was determined through the metabolism of H_2_O_2_ to water, involving concomitant oxidation of reduced glutathione to its oxidized form glutathione disulfide^63^. GR activity was measured by catalysing the transformation of GSSG to reduced GSH with the concomitant oxidation of NADPH to NADP^+^^69^. The glutathione group of enzymes were measured at an absorbance of 340 nm. The peroxidative damage to lipids that occurs with reactive oxygen species generation and results in the production of malondialdehyde was assessed by the determination of TBARS. Malondialdehyde was determined by the thiobarbituric acid and was measured at an absorbance of 532 nm^70^. Micronuclei assay was assessed for genotoxicity. The blood smears were treated with methanol for 10 min to fix the cells and left to dry before staining cells with 5% Giemsa for 30 min. Determination of genotoxicity accounted for the total micronuclei occurrences recorded for 2000 erythrocytes per fish specimen^71^.

### Microplastics

Extraction techniques for the removal of MPs from the gastrointestinal system (foregut to the hindgut) were done following Avio et al.^72^. In summary, a NaCl hypersaline (1.2 g/cm^3^) solution was added at three times the volume of the gastrointestinal system and was stirred and decanted twice for ten minutes, followed by filtration through a 63 µm sieve. The filtrate was transferred from the sieve into a 500 mL beaker and digestion of the suspended organic matter was performed by adding 30 mL of hydrogen peroxide (15%) to the sample before incubation at 60 °C for 24 h. The dried samples were resuspended with distilled water and filtered again through 63 µm sieve. Blank samples were analysed concomitantly with field samples, to gauge any potential cross-contamination. Identification of all plastic particles (different sizes and form type) was performed under a dissecting microscope (Olympus SZ-ST) at 10× magnification and an Infinity 1 camera whose images were processed through the Infinity Analyse software. All MPs larger than 0.5 mm were classified into fragment, film, fibre, and microbead based on their visual aspect^73^.

### Statistical analyses

Seasonal differences in K, HSI, PAH metabolites and MPs were tested using non-parametric Wilcoxon-Mann–Whitney (WMW) test at a 5% significant level. All statistical tests were performed using the R software^74^.

### Ethical statement

Fish specimens and data were collected according to the procedures and protocols approved by the University Research Ethics Committee (UREC) of the University of the South Pacific, in accordance with the policies and guideline stipulated in the Animal Research Ethics Handbook^75^. The processes and procedures of live specimen handling and field transportations were in compliance with Fiji’s regulation of animal protection as outlined in the Protection of Animals Act 1954^76^. Euthanasia method on specimens was consistent with the commonly accepted norms of veterinary best practice^77^.

## Results

A total of 53 specimens of thumbprint emperor were caught and sampled; 31 from the dry season and 22 from the wet season. Mean total weight was 185.5 g (SE = 12.9, range = 86.1–305.5 g) in the dry season and 212.9 g (SE = 13.2, range = 139.6–315.8 g) in the wet season. Average total length was 22.1 cm (SE = 0.7, range = 17.0–27.7 cm) in the dry season and 24.4 cm (SE = 0.5, range = 21.0–28.6 cm) in the wet season. The yearly average total weight was 196.9 g (SE = 9.5, range = 86.1–315.8 g), while the average total length was 23.1 cm (SE = 0.5, range = 17.0–28.6 cm).

### Biomarkers

K was significantly lower (WMW test: Z = 2.02, *p* = 0.042) in samples from the wet season (mean ± SE = 1.42 ± 0.03) compared to the dry season (1.65 ± 0.04), while there was no significant difference in HSI (dry season = 0.82 ± 0.12; wet season = 0.47 ± 0.06; WMW test: Z = 1.21, *p* = 0.228). PAHs values (Table 1) were not significantly different among the two seasons (WMW test: benzo[a]pyrene: Z =  − 1.04, *p* = 0.303; 1-hydroxypyrene: Z = 0.40, *p* = 0.689; phenanthrene Z = 1.83, *p* = 0.066; naphthalene: Z = − 1.03, *p* = 0.308). The observed biochemical responses (Table 2) were also not significantly different between the two seasons (WMW tests: EROD: Z = 0.17, *p* = 0.865; GST: Z =  − 0.41, *p* = 0.685; GPX: Z =  − 0.34, *p* = 0.741; GR: Z =  − 0.83, *p* = 0.414; LPO: Z =  − 1.82, *p* = 0.680; micronuclei assay: Z = 0.71, *p* = 0.407).

**Table 1:** Concentration of polycyclic aromatic hydrocarbon metabolite types (means ± SE) in thumbprint emperor sampled at Vueti Navakavu LMMA during the dry (N = 31 fish) and wet (N = 22 fish) seasons in Fiji.

Season	Benzo[a]pyrene type (mg/L)	1-Hydroxypyrene type (mg/L)	Phenanthrene type (mg/L)	Naphthalene type (mg/L)	Total PAHs (mg/L)
Dry	0.19 ± 0.02	0.35 ± 0.05	88.37 ± 10.28	51.84 ± 4.51	138.57 ± 13.85
Wet	0.24 ± 0.05	0.28 ± 0.05	57.23 ± 6.41	62.58 ± 7.41	110.93 ± 12.71
Total	0.21 ± 0.02	0.32 ± 0.04	75.74 ± 6.89	56.30 ± 4.12	127.10 ± 9.85

**Table 2:** Hepatic ethoxyresorufin *O*-deethylase (EROD), glutathione *S*-transferase (GST), glutathione peroxidase (GPX), glutathione reductase (GR), lipid peroxidation (LPO) and micronuclei assay in thumbprint emperor sampled at Vueti Navakavu LMMA in the dry (N = 31) and wet (N = 22) seasons in Fiji, expressed as mean ± SE.

Season	EROD (nmol/min/mg protein)	GST (nmol/min/mg protein)	GPX (nmol/min/mg protein)	GR (nmol/min/mg protein)	LPO (nmol/mg protein)	Micronuclei assay (per 2000 erythrocytes)
Dry	1.13 ± 0.24	58.17 ± 10.66	11.33 ± 2.84	24.87 ± 4.12	0.99 ± 0.12	0.65 ± 0.13
Wet	0.95 ± 0.23	41.45 ± 5.19	10.30 ± 1.64	27.53 ± 1.75	2.36 ± 0.44	0.59 ± 0.18
Total	1.05 ± 0.17	51.23 ± 6.69	10.90 ± 1.80	25.97 ± 2.57	1.79 ± 0.28	0.62 ± 0.10

### Microplastics

A total of 206 MPs were found. All sampled fish contained at least one MP, with three fish having 10 to 18 MP (Figure S1). On average, the ingestion levels were not significantly higher in fish from the wet season compared to those from the dry season (mean ± SE = 4.7 ± 0.9 MP/fish, 3.3 ± 1.3 MP/fish, respectively; WMW test: Z = − 0.62, *p* = 0.537). The majority of the MP found was less than 1.0 mm in size (Table 3). In particular, MPs ranging 0.1 to 0.4 mm made up 20% of the samples in the dry season and 50% of the samples in the wet season, while MPs ranging 0.5 to 0.9 mm made up 60% of the samples in the dry season and 38% in the wet season.

**Table 3:** Abundance (mean ± SE) and percent contribution (%) of MPs size classes found in thumbprint emperor sampled at Vueti Navakavu LMMA in the dry and wet seasons in Fiji.

Season	0.1–0.4 mm (%)	0.5–0.9 mm (%)	1.0–1.4 mm (%)	1.5–1.9 mm (%)
Dry	0.3 ± 0.1 (20%)	0.7 ± 0.2 (60%)	1.1 ± 0.1 (12%)	1.7 ± 0.1 (8%)
Wet	0.4 ± 0.1 (50%)	0.7 ± 0.2 (38%)	1.0 ± 0.1 (9%)	1.7 ± 0.1 (3%)
Total	0.4 ± 0.0	0.7 ± 0.0	1.2 ± 0.1	1.8 ± 0.0

**Figure 2:**
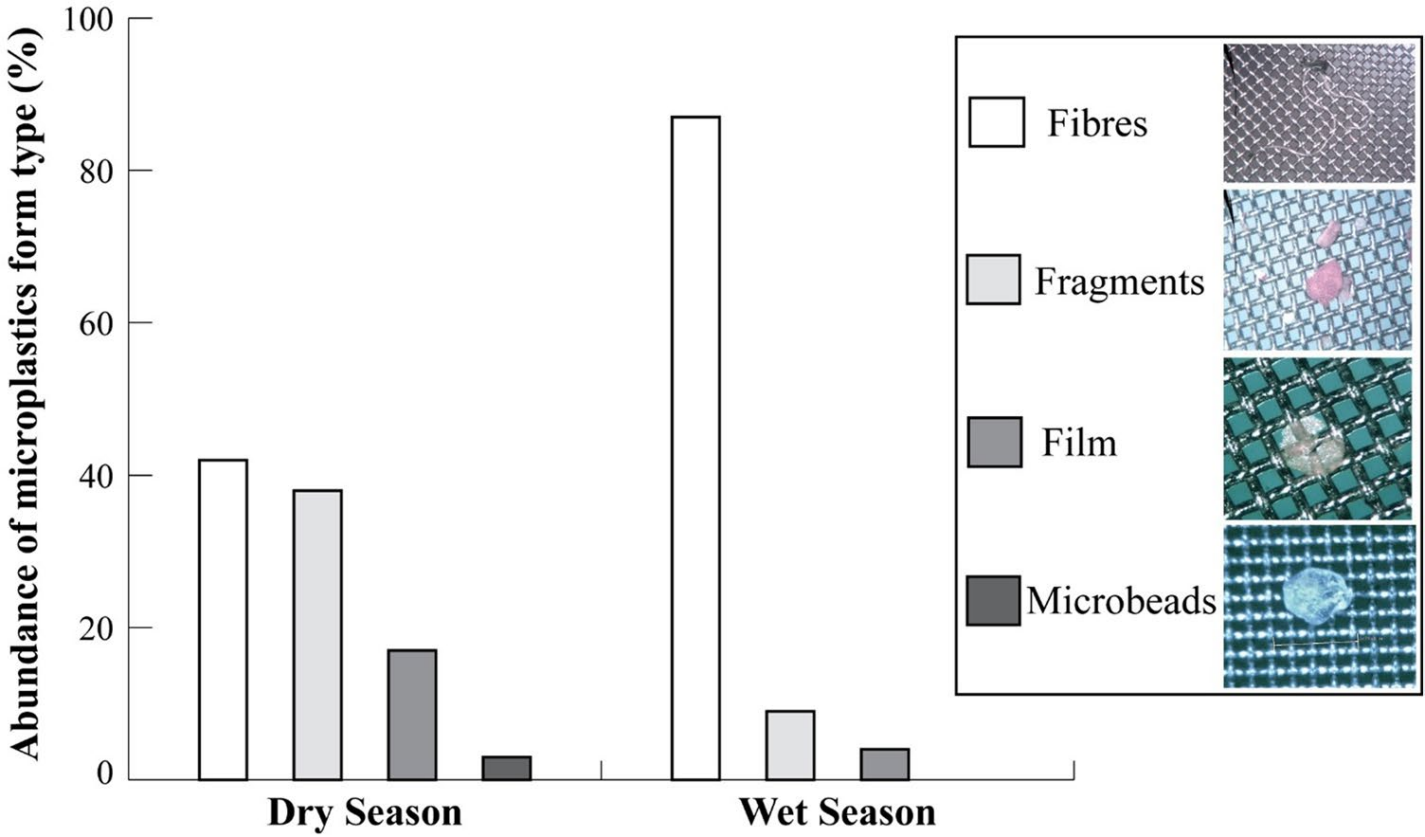
Form types of microplastic pieces found in the gastrointestinal system of thumbprint emperor sampled at Vueti Navakavu LMMA in the dry (N = 103 MPs) and wet (N = 103 MPs) seasons in Fiji.

MP form type was dominated by fibres in the wet season (87% of 103 samples), while in the dry season a similar abundance of fibres and fragments (42% and 38%, respectively) was recorded (Fig. 2). Films had an overall lower abundance, although it was significantly higher in the dry season than in the wet season (0.6 ± 0.2 MP/fish and 0.2 ± 0.1 MP/fish, respectively; WMW test: Z = 2.38, *p* = 0.012). No significant difference was found in the abundance of fibres (dry season = 4.1 ± 0.9 MP/fish, wet season = 1.4 ± 0.3 MP/fish; WMW test: Z =  − 2.01, *p* = 0.435) and fragments (dry season = 1.3 ± 0.2 MP/fish, wet season = 0.4 ± MP/fish; WMW test: Z = 1.73, *p* = 0.081) found in the fish. Microbeads had the lowest abundance and were found in the dry season only.

## Discussion

This study demonstrates a low level of PAHs and MPs in the gastrointestinal tract of mature-sized thumbprint emperor of Vueti Navakavu LMMA in Fiji, South Pacific, both in the wet and dry seasons of the year. All the sampled specimens were of mature size^78^; the observed differences in K were thus most likely resulting from the reproductive activity. Indeed, the thumbprint emperor reportedly reproduces in the wet season^79^. This is further supported by a lower HSI in the wet season, suggesting a lower general stored energy. Variations in energy reserves based on the two physiological biomarkers can also occur as a result of recent feeding habits that can affect the liver size and bodyweight of the fish^80^. To identify whether reproduction or feeding behaviour resulted in the observed differences, we suggest identification of the maturation stage of the gonads via histology^81^ in future ecotoxicological studies.

The potential effects of PAHs on thumbprint emperor are of interest because the species, during its life cycle, occupies key positions in the food web^82^, and also because it is commonly consumed by the villagers of the local community^83^. PAHs enter fishes via food intake and during respiration, are then transported through the bloodstream, and adsorbed to lipid-rich tissues such as liver, muscle tissues, and gonads^84^. The concentration of PAHs found in fishes is normally higher than in the environment from which they were taken^8^. PAH metabolites were present in the bile of all thumbprint emperor sampled, ranging from 0.21 mg/L (benzo[a]pyrene type) to 75.74 mg/L (phenanthrene type) and, altogether, PAHs summed up to 127.10 mg/L. Compared with values found in the Atlantic cod (*Gadus morhua*) sampled from a PAH-polluted site in western Norway, where the lowest concentration was 66 mg/L (benzo[a]pyrene) and the highest concentration was 2704 mg/L (naphthalene)^85^, the ones found in the locally managed areas likely represent a low level of PAH pollution. However, coastal communities that tend to consume large quantities of fishes^86^ could be at greater risk to health issues like growth reduction, endocrine alterations^87^ and gastrointestinal infections^88^ due to PAH bioaccumulation and biomagnification. Monitoring of biliary biotransformation products permits detection of polar metabolites, which are proven to be sensitive PAH exposure markers, possibly two orders of magnitude more sensitive than tissue parent compound levels and is therefore ideal in monitoring and environmental risk assessment studies^89^. The majority of the fishes in Fijian rural areas are sourced locally from nearshore and coastal areas^90^ and in Vueti Navakavu, 88% of all households are involved in fishing activities for income and subsistence purposes^91^. Vethaak et al.^92^ found that marine areas close to industrial harbours have a gradual increase in PAH pollution over time. Site-specific pre-impact assessments are necessary to accurately evaluate before-after, control, impact (BACI) effects^93^. The values identified in this study represent a baseline against which comparison of future levels of biliary PAHs in the thumbprint emperor can be performed to assess environmental improvements in pollution reduction resulting from new policies and procedures.

The hepatic expressions for phase I and II biotransformation showed a total EROD and GST activity of 1.05 nmol/min/mg protein and 51.23 nmol/min/mg protein, respectively. A problem that frequently occurs in field studies with biomarkers is the difficulty of finding suitable reference values, which can be overcome with range-value comparisons of wild species to laboratory-conditioned species^94^. In this case, the Mozambique tilapia (*Oreochromis mossambicus*) exposed to phenanthrene concentration below 4.0 μg/g was found to have EROD activity of 5.4 nmol/min/mg protein, and to exhibit sublethal hepatotoxicity^95^. These biochemical values of EROD response are 5.1 times higher than those found in thumbprint emperor in this study, although without experimental studies in laboratorial conditions the sublethal consequences of this activity cannot be excluded. Compared with literature, GST activity in the present study was much lower than in two flatfishes (English sole *Parophrys vetulus*: 16-fold; starry flounder *Platichthys stellatus*: 44-fold) captured from a contaminated site in Puget Sound, Washington, and was related to higher activation and lower detoxification ability of PAHs resulting in hepatic neoplasms and putatively preneoplastic lesions^96^. Although the types of biotransformation reactions are similar between fishes, differences exist in the metabolic handling of chemicals; particularly reactions rates, the relative contribution of a given pathway, and the products formed^97^. From a functional viewpoint, biotransformation reactions can significantly influence the biological properties of chemicals, depending on the nature of the reaction and the rate at which it occurs^98^. In the case of the thumbprint emperor from Vueti Navakavu, phase I and II biotransformation activity were observed although there was no statistically clear relationship between the two pollutants.

Total activity for GPX and GR responses were 10.90 and 25.97 nmol/min/mg protein, respectively. The reaction of biomarkers GPX and GR is naturally inverse within a biological system^69^. The GPX responses in thumbprint emperor (10.90 nmol/min/mg protein) in the present study was lower than the one (11.89 nmol/min/mg protein) found on sterlet sturgeon (*Acipenser ruthenus*) in a petroleum-polluted site in Novi Sad (Serbia) but higher than the levels reported in a control site (10.30 nmol/min/mg protein) in the same place^99^. Consistently with the expected opposite enzymatic role of GR, the recorded GR activity in thumbprint emperor was 2.4 times higher than the GPX reaction. This is consistent with effective hepatic biochemical responses of the thumbprint emperor under oxidative stress due to oxidative-inducing pollutants, likely as a result of a significant accumulation of hydrogen peroxide^100^. We suggest including variations of antioxidant system response and its regulatory mechanisms under different circumstances (e.g., biological effects from exposure to heavy metals) in further studies, which would provide a valuable assessment of seafood fish quality and health risks from consumption.

A low level of biological damages was identified by LPO concentrations, whereby the levels found in the thumbprint emperor in the present study were 17 times lower than those found in spotted snakehead (*Channa* *punctatus*) in Aligarh, India, which were exposed to wastewater and, as a result, reported clear membrane damage. LPO concentrations mainly depend on the availability of polyunsaturated fatty acids and the antioxidants defences^101^. Consistently, the micronuclei assay in thumbprint emperor showed the very low occurrence of abnormal nuclei formation in erythrocytes (0.03%) compared to genotoxic findings on Nile tilapia (*Oreochromis niloticus*) (0.95%) captured from a heavy metals-polluted site in India^102^.

MPs were found in thumbprint emperors in both seasons, and the most abundant were smaller than 1.0 mm. The size of MPs may be a key factor in determining the range of animals that ingest them^103^, as well as the retention rate; for example, retention in the gastrointestinal system of spiny Chromis (*Acanthochromis polyacanthus*) increased (with a maximum of 2102 small particles) when the MPs size was reduced from 2 mm to 0.3**–**0.125 mm^104^. MPs cause oxidative damages in gills, muscle, and an increase in neurotoxic responses of fishes^105^. Low exposure to MPs is supported by both the low numbers of MPs found in thumbprint emperor as well as the low level of oxidative stress identified in the present study. Studies of MPs in seafood fish^106–108^ have also found that some pollutants like PAHs have a greater affinity to MPs than to water, and as a result MPs ingestion by seafood fishes likely have equal exposure to PAH intake (including other chemical pollutants), leading to bioaccumulation and biomagnification. It is for this reason that MPs and PAHs is recommended herein to be included in monitoring programmes in Fiji and other Pacific Island countries. While this baseline study observes the presence of MPs and PAHs in the thumbprint emperor, it only serves as a reference point for future pollution studies in seafood fishes in Fiji, and does not report risks or make recommendations from a human health perspective.

The PAHs and MPs pollutants quantified in this study represent a baseline for Vueti Navakavu LMMA, and indeed despite not having statistical significance, the interpretation of biologically relevant effects and efforts should be carefully considered. It is generally more valuable in ecotoxicology studies to know about the magnitude and certainty of an effect as well as its probability to occur, to ultimately evaluate its biological relevance^109^. Gagnon and Hodson^110^ demonstrated that biomarkers may vary in proportion to the extent of exposure to pollutants, and that individual studies might discover different magnitudes and directions of biomarker responses according to the specific situation investigated. In this study, the use of ecotoxicological tools (i.e. biomarkers) allows for direct assessment of the health of the thumbprint emperor within the LMMA since this approach shifts the focus of the assessment from the agents (pollutants) in the environmental compartments to the target species (biological responses)^111^. The thumbprint emperor, as aforementioned, showed relatively low levels of PAHs exposure and MPs ingestion, including relatively low levels of biological damages shown by ENA and LPO concentrations. The biochemical responses of Phase I and II biotransformation, GPX and GR activity were also comparatively low to studies that were identified. As a baseline for pollutants quantification and biochemical responses, it is important to not exclude the prospect of Vueti Navakavu’s twenty-long years as an LMMA being of some consequence to the health conditions and low pollutants exposure in the thumbprint emperor. Furthermore, this baseline study does not assume a “normal” biological state being defined for the thumbprint emperor, and the observation of pollutants presence and histopathological injuries that were observed in the fish is considered biologically relevant from a toxicological perspective^112^. It is for this reason that future biomonitoring studies is encouraged to build on the baseline information identified herein and employ a suite of biomarkers to bridge knowledge gaps and enable environmental risk assessment programmes to strengthen conservation efforts in marine spaces.

Marine protected areas are legally designated areas where human activities are restricted or managed to ensure sustainability and avoid over-fishing and habitat destruction^113^. Enforcement and management are essential to ensure that marine protected areas fulfil their purpose and do not remain mere polygons on a chart. Moreover, the effectiveness of these areas in protecting the marine and coastal habitat, their resistance and resilience to pollution, and their capacity of providing benefits like ecosystem services and ecological spillover, is related to the size of the MPAs and the place where the MPAs are established^114^.

The Vueti Navakavu LMMA is representative of Fiji’s key coral reef and mangrove coastal habitats, but it is relatively small-sized and its location is a few kilometres on the west of the harbour. Reportedly, the LMMA is threatened by chemical and solid waste pollution from the former Lami Dump and Suva city, both of which are located upwind and up-current^115^. It is important noting that the boundaries of the LMMA have cultural significance and are not ecological boundaries, therefore they are neutral and completely permeable to pollution.

Our study shows the presence of PAHs and MPs in all samples of emperor fish, an important seafood fish. Seasonal differences to the multibiomarker responses and pollutants levels were not statistically significant, which suggests that the threat posed by PAHs and MPs in Vueti Navakavu LMMA is all year round. Emerging pollutants (like MPs) and legacy pollutants (like PAHs) move unrestrictedly beyond conservation systems and boundaries such as LMMAs and MPAs. The baseline biological and pollutants parameters, herein, may provide meaningful insights for future biomonitoring studies in Fiji, with site differences and the inclusion of fish species comparison. We also recommend expanding the range and suite of biomarkers to contribute to enhancing a more holistic baseline information for other marine settings in Fiji and Pacific Island countries.

## Supplementary Information

Supplementary Information.

## Data Availability

All data generated or analysed during this study are included in this published article (and its Supplementary Information files).
