# Supplementary material for: Multibiomarker responses to polycyclic aromatic hydrocarbons and microplastics in thumbprint emperor Lethrinus harak from a South Pacific locally managed marine area
Source: Sci Rep. 2021 Sep 9;11:17991. doi: 10.1038/s41598-021-97448-4 (PMC8429447; doi:10.1038/s41598-021-97448-4)
Supplement: Supplementary file 1 — Supplementary Information. [file 41598_2021_97448_MOESM1_ESM.pdf]

Multibiomarker responses to polycyclic aromatic hydrocarbons and microplastics in thumbprint emperor *Lethrinus harak* from a South Pacific locally managed marine area

Rufino Varea*, Andrew Paris, Marta Ferreira and Susanna Piovano

School of Agriculture, Geography, Environment, Ocean and Natural Sciences, The University of the South Pacific, Suva, Fiji.

*** Corresponding Author**: s11088132@student.usp.ac.fj

Supplementary:

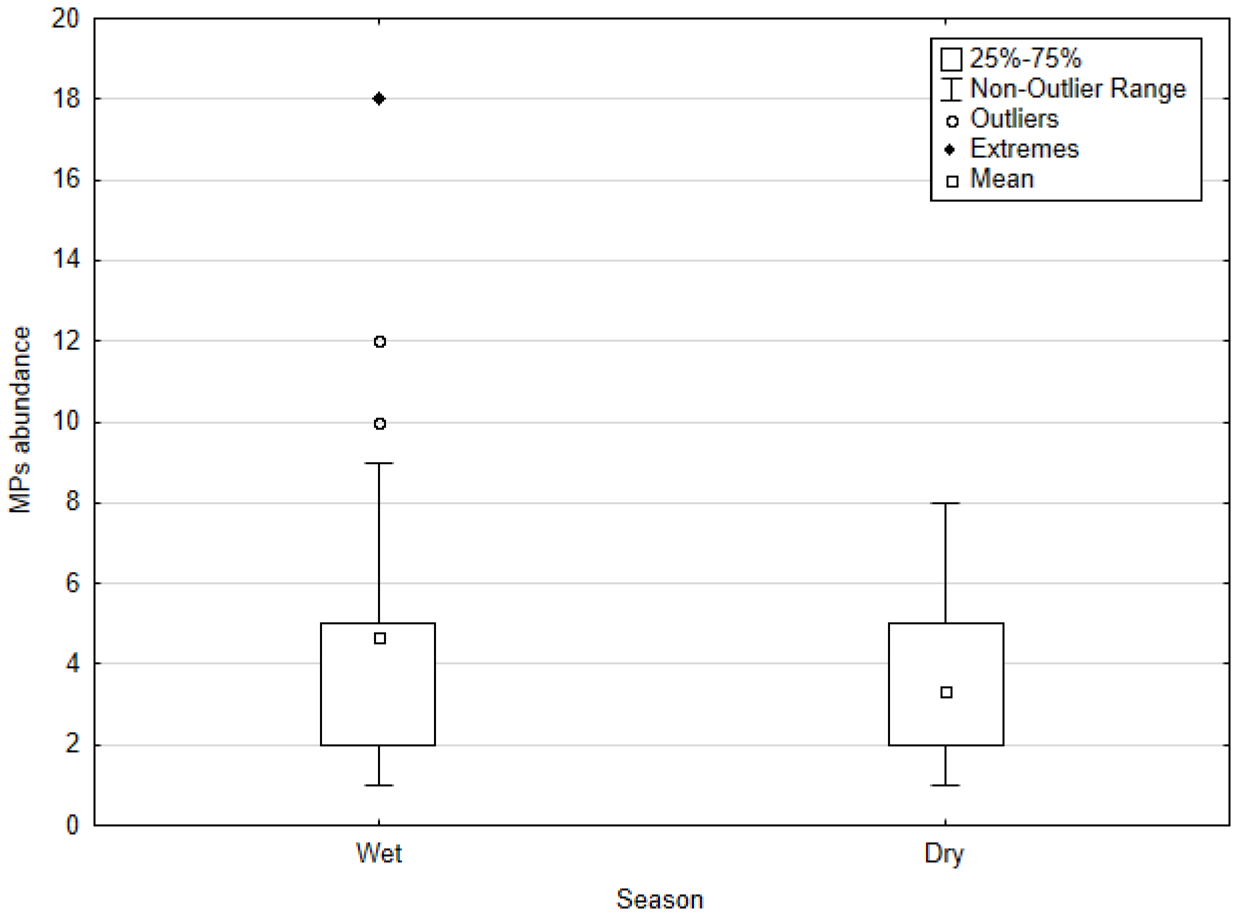

Figure S1: Average abundance of microplastic pieces (MPs) in the gastrointestinal system of *Lethrinus harak* sampled at Vueti Navakavu LMMA in the wet and dry seasons in Fiji.
